# Differential Effect of Polymorphisms on Body Mass Index Across the Life Course of Japanese: The Japan Multi-Institutional Collaborative Cohort Study

**DOI:** 10.2188/jea.JE20190296

**Published:** 2021-03-05

**Authors:** Madoka Iwase, Keitaro Matsuo, Masahiro Nakatochi, Isao Oze, Hidemi Ito, Yuriko Koyanagi, Tomotaka Ugai, Yumiko Kasugai, Asahi Hishida, Kenji Takeuchi, Rieko Okada, Yoko Kubo, Chisato Shimanoe, Keitaro Tanaka, Hiroaki Ikezaki, Masayuki Murata, Toshiro Takezaki, Daisaku Nishimoto, Nagato Kuriyama, Etsuko Ozaki, Sadao Suzuki, Miki Watanabe, Haruo Mikami, Yohko Nakamura, Hirokazu Uemura, Sakurako Katsuura-Kamano, Kiyonori Kuriki, Yoshikuni Kita, Naoyuki Takashima, Masato Nagino, Yukihide Momozawa, Michiaki Kubo, Kenji Wakai

**Affiliations:** 1 Division of Cancer Epidemiology and Prevention, Aichi Cancer Center Research Institute, Nagoya, Japan; 2 Department of Surgical Oncology, Nagoya University Graduate School of Medicine, Nagoya, Japan; 3 Division of Cancer Epidemiology, Nagoya University Graduate School of Medicine, Nagoya, Japan; 4 Department of Nursing, Nagoya University Graduate School of Medicine, Nagoya, Japan; 5 Division of Cancer Information and Control, Aichi Cancer Center Research Institute, Nagoya, Japan; 6 Division of Descriptive Cancer Epidemiology, Nagoya University Graduate School of Medicine, Nagoya, Japan; 7 Department of Preventive Medicine, Nagoya University Graduate School of Medicine, Nagoya, Japan; 8 Clinical Research Center, Saga University Hospital, Saga, Japan; 9 Department of Preventive Medicine, Faculty of Medicine, Saga University, Saga, Japan; 10 Department of General Internal Medicine, Kyushu University Hospital, Fukuoka, Japan; 11 Department of International Island and Community Medicine, Kagoshima University Graduate School of Medical and Dental Sciences, Kagoshima, Japan; 12 School of Health Sciences, Faculty of Medicine, Kagoshima University, Kagoshima, Japan; 13 Department of Epidemiology for Community Health and Medicine, Kyoto Prefectural University of Medicine, Kyoto, Japan; 14 Department of Public Health, Nagoya City University Graduate School of Medical Sciences, Nagoya, Japan; 15 Cancer Prevention Center, Chiba Cancer Center Research Institute, Chiba, Japan; 16 Department of Preventive Medicine, Institute of Biomedical Sciences, Tokushima University Graduate School, Tokushima, Japan; 17 Laboratory of Public Health, University of Shizuoka, Shizuoka, Japan; 18 Faculty of Nursing Science, Tsuruga Nursing University, Fukui, Japan; 19 Department of Public Health, Faculty of Medicine, Kindai University, Osaka, Japan; 20 Laboratory for Genotyping Development, RIKEN Center for Integrative Medical Sciences, Yokohama, Japan; 21 RIKEN Center for Integrative Medical Sciences, Yokohama, Japan

**Keywords:** obesity, body mass index, genome wide association study, polymorphisms

## Abstract

**Background:**

Obesity is a reported risk factor for various health problems. Genome-wide association studies (GWASs) have identified numerous independent loci associated with body mass index (BMI). However, most of these have been focused on Europeans, and little evidence is available on the genetic effects across the life course of other ethnicities.

**Methods:**

We conducted a cross-sectional study to examine the associations of 282 GWAS-identified single nucleotide polymorphisms with three BMI-related traits, current BMI, BMI at 20 years old (BMI at 20), and change in BMI (BMI change), among 11,586 Japanese individuals enrolled in the Japan Multi-Institutional Collaborative Cohort study. Associations were examined using multivariable linear regression models.

**Results:**

We found a significant association (*P* < 0.05/282 = 1.77 × 10^−4^) between BMI and 11 polymorphisms in or near *FTO*, *BDNF*, *TMEM18*, *HS6ST3*, and *BORCS7*. The trend was similar between current BMI and BMI change, but differed from that of the BMI at 20. Among the significant variants, those on *FTO* were associated with all BMI traits, whereas those on *TMEM18* and *HS6SR3* were only associated with BMI at 20. The association of *FTO* loci with BMI remained, even after additional adjustment for dietary energy intake.

**Conclusions:**

Previously reported BMI-associated loci discovered in Europeans were also identified in the Japanese population. Additionally, our results suggest that the effects of each loci on BMI may vary across the life course and that this variation may be caused by the differential effects of individual genes on BMI via different pathways.

## INTRODUCTION

Obesity is a known risk factor for various diseases^1^^–^^3^ and has increased globally in recent decades. Studies in twins and families suggest the existence of genetic factors for obesity.^4^^–^^7^ In recent years, genome-wide association studies (GWASs) have identified genes for common traits and enabled the identification of numerous obesity-related genetic variants.^8^^–^^11^ Among obesity-related traits, body mass index (BMI) is a well-established measure for evaluating obesity,^12^^,^^13^ and BMI-associated GWASs have reported a substantial number of related polymorphisms. Among these studies, the largest population meta-analysis study included 339,224 individuals and identified 97 BMI-associated loci.^14^

One recent GWAS conducted in a Japanese population using data from the BioBank Japan (BBJ) project reported 112 new BMI-associated loci^15^ and indicated genetic differences between European and East Asians. Apart from this study, however, most GWASs have been conducted in participants of European ancestry, and findings in non-Europeans are not currently sufficient.^16^^,^^17^ In addition, not only does BMI vary among ethnicities, but it also changes over the life course. For example, weight changes markedly in adulthood, and weight gain in early to middle adulthood is common.^18^^,^^19^ Numerous BMI-associated GWASs have been conducted using BMI determined at a single point in a person’s life, and it remains unclear at what age BMI is most likely to be affected by genetic variants or how these variants affect weight gain.

Therefore, the aims of this study were to identify BMI-associated loci in the Japanese population and to investigate how genetic factors affect BMI through the life course.

## METHODS

### Study population

The study was conducted using data from the Japan Multi-Institutional Collaborative Cohort (J-MICC) Study. Details of the J-MICC study have been described elsewhere.^20^ Briefly, the J-MICC study was launched in 2005 to investigate gene-environment interactions of lifestyle-related diseases. The subjects of the present study were volunteers aged 35 to 69 years who provided blood samples and information on their lifestyle via a questionnaire. The J-MICC study had recruited 102,145 participants by June 2018. Written informed consent was obtained from all participants. A total of 14,539 participants were randomly selected to be genotyped from a total of 47,163 participants from the 12 original participating sites: Aichi, Chiba, Fukuoka, Kagoshima, KOPS, Kyoto, Okazaki, Sakuragaoka, Saga, Shizuoka-Daiko, Takashima, and Tokushima. Genotyping was performed at the RIKEN Center for Integrative Medicine using a HumanOmniExpressExome-8 v1.2 BeadChip array (Illumina Inc., San Diego, CA, USA). Quality control (QC; described below) was conducted for the remaining 14,539 participants, and 422 participants whose genotyping data did not meet the QC filters were excluded. Among the remaining 14,091 participants, 32 were excluded due to a lack of questionnaire data.

In addition, participants were excluded from the study if they had missing or extreme values for self-reported weight at baseline (<30 kg), weight at age 20 (<30 kg), height (<130 cm), and current BMI (>60) and BMI at 20 (>60) (*n* = 413). Patients with cancer at baseline were also excluded from the analysis (*n* = 2,114). Fifty-four patients with both being outlier and cancer were included in these numbers, so a total of 2,473 participants were excluded. The remaining 11,586 subjects were analyzed (eFigure 1).

### Quality control and genotype imputation

Participants with inconsistent baseline information on sex between the questionnaire and genotyping results were excluded (*n* = 26). The identity-by-descent method implemented in PLINK software^21^^,^^22^ identified 388 close relationship pairs (pi-hat >0.1875); one sample of each pair was excluded. According to principal component analysis with the 1000 Genomes reference panel (phase 3),^23^ 34 subjects with non-Japanese ancestry were detected and excluded.^24^ The remaining samples met the sample-size genotype call rate criterion (≥0.99). Single nucleotide polymorphisms (SNPs) with genotype call rate <0.98, a Hardy-Weinberg equilibrium extract test *P*-value <1 × 10^−6^, a minor allele frequency of <0.01, or a departure from the allele frequency computed from the 1000 Genomes Project phase 3 EAS samples were removed. Non-autosomal SNPs were also removed. This QC filtering left 14,091 individuals. Genotype imputation was performed using SHAPIT2^25^ and Minimac3^26^ software based on the 1000 Genomes reference panel (phase 3). After genotype imputation, we excluded variants with an imputation quality score *r*^2^ < 0.3, resulting in 12 617 547 variants (J-MICC data set ver. 20180111).

### Definition of BMI

BMI was calculated by dividing body weight in kilograms by the square of height in meters. We examined three BMI traits: (i) current BMI, defined as the BMI calculated based on the self-reported current weight and current height; (ii) BMI at 20, defined as the BMI calculated based on the self-reported weight at 20 years old and current height; and (iii) BMI change (per year), defined as (current BMI − BMI at 20)/(age − 20).

### Measurement of lifestyle factors

Lifestyle factors, including smoking status and dietary intake, and medical information were evaluated using a self-administered questionnaire. Information on dietary intake of total energy was estimated using a food frequency questionnaire containing 47 food items^27^ based on the Standard Tables of Food Composition in Japan.^28^

### SNP selection

We selected BMI-associated SNPs based on a previous Japanese study and GWAS catalog.^29^ First, we selected SNPs shown to be associated with BMI in a previous BBJ study that included Japanese-specific loci.^15^ We extracted loci in both trans-ethnic and Japanese specific, which are total of 261 significant loci reported by the BBJ study as the first group. Among these, SNPs on the X chromosome (*n* = 5) were excluded. Second, we selected 35 SNPs reported to be associated with “childhood body mass index” using the GWAS catalog^29^ as the second group. Third, we selected 45 SNPs reported to be associated with “longitudinal BMI measurement” in the GWAS catalog^29^ as the third group. Because 10 SNPs were duplicated in two or more groups, the total number of extracted SNPs was actually 326. We excluded SNPs with no available genotyping information in our J-MICC database (*n* = 10) and additionally excluded SNPs with a MAF less than 5% (*n* = 34), leaving 282 loci for analysis (eFigure 2, eTable 1, and eTable 2). Linkage disequilibrium (LD) was evaluated between SNPs on the same chromosome that showed a significant association with BMI by using both LDlink^30^ based on 1000 Genomes Project phase 3 (JPT) and our dataset. We defined two loci are in LD when *R*^2^ is above 0.80.

### Statistical analysis

The relationship between each BMI-related phenotype and SNPs was analyzed using a crude and multivariable linear regression model to calculate the regression coefficient (β) and to evaluate the significance of the association (*P*-value). The adjusted model adjusted for age at baseline (continuous), age-squared (continuous), sex (men or women), birth year (continuous), and first five principal components (PCs) (continuous) in current BMI; sex, birth year, and five PCs in BMI at 20; age, age-squared, sex, birth year, first five PCs, and BMI at 20 in BMI change. At this level, we screened SNPs based on threshold *P*-values. Next, to evaluate the robustness of the association with the selected SNPs, we conducted a sex-stratified analysis and never-smokers only analysis. We also assessed heterogeneity among sex. To examine the potential effect of dietary energy intake on current BMI, we added total dietary energy intake to the adjusted model. Energy intake was defined as the individual total dietary energy intake divided by current weight.

All statistical analyses were conducted using Stata version 15.1 software (Stata Corp., College Station, TX, USA). *P*-values in both main analysis and stratified analyses were considered statistically significant at *P* < (0.05/282 = 1.77 × 10^−4^) following Bonferroni correction. We considered *P*-value <0.05 as significant only in assessing heterogeneity among sex.

Heritability analysis for each of three BMI phenotypes was performed with the use of genomic restricted maximum likelihood (GREML) method implemented in GCTA software.^31^ The analysis estimates the percentage of phenotypic variance explained by common SNPs. To estimate the heritability, we used the data set comprising the 11,586 subjects (5,169 males and 6,417 females) adopted for the association analysis as well as the 570,162 directly genotyped SNPs used for imputation. The phenotypic values for current BMI were adjusted for age at baseline, age-squared, birth year, sex, and first five principal components. The phenotypic values for BMI at 20 were adjusted for birth year, sex, and first five principal components. We applied two types of model to estimate the heritability of BMI change. The covariates in model 1 comprised age at baseline, age-squared, birth year, sex, and first five principal components. The covariates in model 2 included those of model 1 as well as BMI and BMI at 20. The estimation of the heritability was also performed using male and female subjects, separately. To estimate the genetic correlations among three BMI phenotypes, bivariate GREML^32^ was conducted using GCTA software. The estimate of heritability by GCTA is composed of two steps. At first, the genetic relationship matrix (GRM) between pairs of individuals was estimated from a set of SNPs using the GCTA option “--make-grm --thread-num 40”. This process is computationally intensive, so the process was performed by multi-thread computing with 40 CPU cores. Next, the heritability was estimated using the estimated GRM and the GCTA option “--reml”. We calculated the genetic correlations employing the same data sets, which were used to estimate the heritability. The genetic correlation was calculated using the same GRM and the GCTA option “--reml-bivar”. The phenotypic values for each of current BMI and BMI change were adjusted for age at baseline, age-squared, birth year, sex, and first five principal components. The phenotypic values for BMI at 20 were adjusted for birth year, sex, and first five principal components.

## RESULTS

The baseline characteristics of the study subjects are shown in Table 1. All participants were Japanese and the 12 study sites were widely distributed throughout the western part of Japan. The study comprised 5,169 men (44.6%) and 6,421 women (55.4%), with an average age of 54.1 years. The averages of respective BMI phenotypes were as follows: 23.1 kg/m^2^ in current BMI, 20.9 kg/m^2^ in BMI at 20, and 0.069 kg/m^2^/year in BMI change.

**Table 1.** Baseline characteristics of study subjects

Number of subjects	11,586	

Age, years, mean (SD)	54	(9.4)

Sex, *n* (%)		
Male	5,169	(44.6)
Female	6,417	(55.4)

Smoking status, *n* (%)		
Never smoker	6,865	(59.3)
Ever smoker		
Former smoker	2,331	(20.1)
Current smoker	2,382	(20.6)
Unknown	8	(0.1)

Area, *n* (%)		
Chiba	845	(7.3)
Aichi Cancer Center	695	(6.0)
Okazaki	956	(8.3)
Shizuoka+Daiko	1,883	(16.3)
Takashima	457	(3.9)
Kyoto	958	(8.3)
Fukuoka	715	(6.2)
Saga	1,753	(15.1)
Kagoshima	1,085	(9.4)
Tokushima	641	(5.5)
KOPS	1,112	(9.6)
Shizuoka/Sakuragaoka	490	(4.2)

Birth year, mean (SD)	1,953	(10.1)

BMI phenotype, kg/m^2^, mean (SD)		
Current BMI	23.1	(3.3)
BMI at 20	20.9	(2.4)
BMI change	0.069	(0.098)

BMI, body mass index; KOPS, Kyusyu Okinawa Population Study; SD, standard deviation.

**Figure 1.**
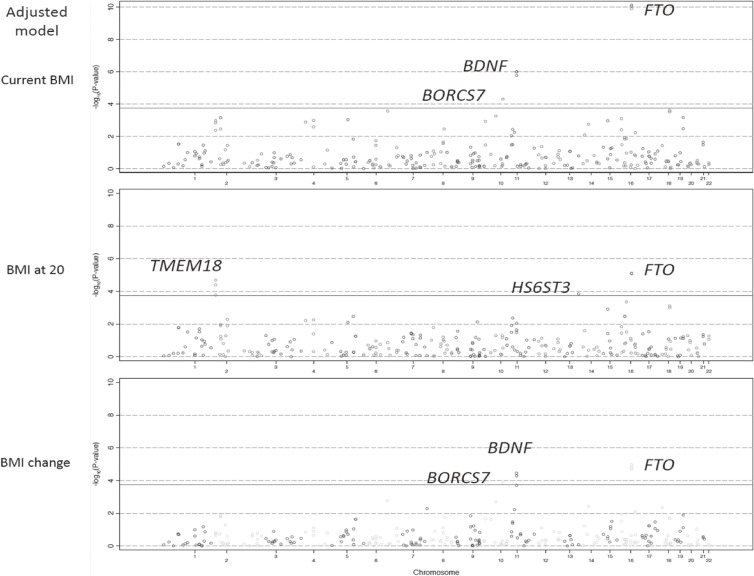
Manhattan plots of the association between candidate loci and each BMI phenotype in the adjusted model. The X-axis represents chromosomal position and the Y-axis represents −log_10_(*P*-value). The grey solid horizontal line indicates significance level (*P* = 1.77 × 10^−4^).

Among the 282 candidate loci, we identified a total of 11 SNPs with a statistically significant association with one or more BMI phenotypes in the adjusted analyses after applying the Bonferroni threshold. Table 2 shows the allele frequency of the significant SNPs and β estimates for the association with BMI. Among these loci, which were located near transmembrane protein 18 (*TMEM18*) (rs939584, rs13021737, and rs4854349), in brain-derived neurotrophic factor (*BDNF*) (rs11030100, rs6265, and rs11030104), and fat mass and obesity-associated gene (*FTO*) (rs1421085, rs11642015, rs1559302) showed LD with *R*^2^ ≥ 0.90 in both our dataset and LDlink (one locus on chromosome 16 (rs1559302) was not available in LDlink). In the non-stratified adjusted model, there was a significant association of 7 loci identified in the current BMI, 6 identified in the BMI at 20, and 6 identified in the BMI change. The allele frequency at each locus was consistent with that in the Japanese population in the 1000 Genome project (phase 3) database.^33^ Loci located in *FTO* were significantly associated with all BMI phenotypes, while loci located near *TMEM18* and *HS6ST3* were only associated with BMI at 20. Loci near *TMEM18* and in *BDNF* were inversely associated, and loci in or near the remaining genes showed a positive association with each of the BMI phenotypes. Figure 1 shows the Manhattan plots for each BMI phenotype. We failed to identify any of the newly identified loci reported in a previous Japanese study among the significant SNPs in our adjusted models.^15^

**Table 2.** Eleven identified SNPs associated with any of the BMI phenotypes

SNP	Chr.^a^	Position (bp)^a^	REF/ALT	Effect allele frequency^b^	Genotyped	Imputation quality score	Candidate gene(s)	Current BMI			BMI at 20			BMI change			Consistency for former reports^g^

								Adjusted^d^			Adjusted^e^			Adjusted^f^			

								β^b^	SE	*P*-value^c^	β^b^	SE	*P*-value^c^	β^b^	SE	*P*-value^c^	
rs939584	2	621558	C/T	0.898	+	1.000	*LOC105373352*, *TMEM18*	−0.220	0.067	1.07E-03	−0.217	0.051	**2.03E-05**	−0.003	0.002	1.52E-01	−
rs13021737	2	632348	A/G	0.898	−	0.993	*LOC105373352*, *TMEM18*	−0.214	0.067	1.48E-03	−0.208	0.051	**4.02E-05**	−0.003	0.002	1.77E-01	−
rs4854349	2	647861	T/C	0.895	−	0.996	*LOC105373352*, *TMEM18*	−0.191	0.067	4.26E-03	−0.190	0.051	**1.66E-04**	−0.002	0.002	2.30E-01	−
rs4409766	10	104616663	T/C	0.295	−	0.996	*BORCS7-ASMT*	0.181	0.045	**4.96E-05**	0.036	0.034	2.93E-01	0.005	0.001	**1.38E-04**	+
rs11030100	11	27677586	G/T	0.403	−	0.963	*BDNF-AS*	−0.199	0.042	**1.68E-06**	−0.082	0.031	8.95E-03	−0.005	−0.003	1.96E-04	+
rs6265	11	27679916	C/T	0.403	−	0.996	*BDNF*	−0.203	0.041	**1.00E-06**	−0.071	0.031	2.26E-02	−0.005	0.001	**5.15E-05**	+
rs11030104	11	27684517	A/G	0.402	−	0.963	*BDNF-AS*	−0.203	0.042	**1.01E-06**	−0.067	0.031	3.18E-02	−0.005	0.001	**3.51E-05**	Not shown^H^
rs1927790	13	96922191	T/C	0.420	+	1.000	*HS6ST3*	0.057	0.041	1.64E-01	0.119	0.031	**1.39E-04**	0.000	0.001	8.34E-01	+
rs1421085	16	53800954	T/C	0.196	−	0.993	*FTO*	0.335	0.051	**1.24E-10**	0.173	0.039	**8.02E-06**	0.007	0.002	**2.04E-05**	+
rs11642015	16	53802494	C/T	0.196	−	0.963	*FTO*	0.334	0.051	**7.36E-11**	0.174	0.039	**8.41E-06**	0.007	0.002	**1.08E-05**	+
rs1558902	16	53803574	T/A	0.195	−	0.993	*FTO*	0.331	0.051	**8.65E-11**	0.174	0.039	**7.69E-06**	0.006	0.002	**1.59E-05**	+

ALT, alternative allele; BMI, body mass index; bp, base pair; Chr., chromosome; REF, reference allele; SE, standard error; SNP, single nucleotide polymorphism.

^a^Positions are based on Human Genome version 19 (hg19), build 37.

^b^Alternative alleles were treated as effect alleles.

^c^Significant *P*-values (*P* ≤ 1.77E-04) are shown in bold.

^d^Adjusted model in current BMI were adjusted for age (continuous), age-squared (continuous), sex (male or female), birth year (continuous), and the first five principal components (continuous).

^e^Adjusted model in BMI at 20 were adjusted for sex (male or female), birth year (continuous), and the first five principal components (continuous).

^f^Adjusted model in BMI change were adjusted for age (continuous), age-squared (continuous), sex (male or female), birth year (continuous), the first five principal components (continuous), and BMI at 20 (continuous).

^g^If the direction of effect allele in initially extracted BMI-related traits was consistent with that of former reports, “+”, if not, “−” is in the column.

^h^The results of estimates were not shown, but only *P*-values were shown in former reports.

In sex-stratified analysis, several loci showed sex-specific association between BMI and genotype, but they did not show any heterogeneity in the 11 significant SNPs (eTable 3). In addition, to eliminate the disease-related weight change which derived from diabetes, we performed same analysis as original in current BMI with excluding participants with history of diabetes mellitus. The tendency of association between current BMI and significant SNPs were similar even after excluding those with diabetes (eTable 4). Given that smoking behavior has a strong effect on body weight,^34^^–^^36^ we conducted an analysis that focused on never smokers. In these subjects, the significant SNPs were similar to those in the adjusted models for the entire population (Table 3). We also examined the effect of food intake by adjusting for energy intake in the analysis of 7 significant loci (located in 3 genes) identified in the current BMI. After adjusting for energy intake, polymorphisms in *FTO* remained significant SNPs (Table 4).

**Table 3.** Eleven identified SNPs associated with any of the BMI phenotypes in never-smokers

SNP	Chr.^a^	Position (bp)^a^	REF/ALT	Candidate gene(s)	Current BMI			BMI at 20			BMI change		
		
					Adjusted^d^			Adjusted^e^			Adjusted^f^		

					β^b^	SE	*P*-value^c^	β^b^	SE	*P*-value^c^	β^b^	SE	*P*-value^c^
rs939584	2	621558	C/T	*LOC105373352*, *TMEM18*	−0.287	0.088	1.03E-03	−0.246	0.065	**1.60E-04**	−0.006	0.003	3.12E-02
rs13021737	2	632348	A/G	*LOC105373352*, *TMEM18*	−0.288	0.087	9.63E-04	−0.254	0.065	**9.05E-05**	−0.005	0.003	3.40E-02
rs3800229	6	108996963	G/T	*FOXO3*	−0.254	0.063	**5.96E-05**	−0.087	0.047	6.37E-02	−0.006	0.002	1.25E-03
rs143665886	7	115368366	T/C	*SNORA25B, TFEC*	0.037	0.053	4.85E-01	0.151	0.039	**1.31E-04**	−0.001	0.002	5.27E-01
rs4409766	10	104616663	T/C	*BORCS7-ASMT*	0.236	0.058	**4.26E-05**	0.075	0.043	8.11E-02	0.006	0.002	3.72E-04
rs11030100	11	27677586	G/T	*BDNF-AS*	−0.266	0.054	**7.93E-07**	−0.117	0.040	3.50E-03	−0.006	0.002	**1.77E-04**
rs6265	11	27679916	C/T	*BDNF*	−0.265	0.054	**8.14E-07**	−0.097	0.040	1.49E-02	−0.006	0.002	**8.03E-05**
rs11030104	11	27684517	A/G	*BDNF-AS*	−0.261	0.054	**1.14E-06**	−0.090	0.040	2.37E-02	−0.006	0.002	**6.35E-05**
rs1421085	16	53800954	T/C	*FTO*	0.358	0.067	**7.39E-08**	0.164	0.050	9.61E-04	0.007	0.002	**1.41E-04**
rs11642015	16	53802494	C/T	*FTO*	0.361	0.067	**5.74E-08**	0.163	0.050	1.00E-03	0.008	0.002	**1.07E-04**
rs1558902	16	53803574	T/A	*FTO*	0.365	0.067	**4.45E-08**	0.164	0.050	9.24E-04	0.008	0.002	**9.54E-05**

ALT, alternative allele; BMI, body mass index; bp, base pair; Chr., chromosome; REF, reference allele; SE, standard error; SNP, single nucleotide polymorphism.

^a^Positions are based on Human Genome version 19 (hg19), build 37.

^b^Alternative alleles were treated as effect alleles.

^c^Significant *P*-values (*P* ≤ 1.77E-04) are shown in bold.

^d^Adjusted model in current BMI were adjusted for age (continuous), age-squared (continuous), sex (male or female), birth year (continuous), and the first five principal components (continuous).

^e^Adjusted model in BMI at 20 were adjusted for sex (male or female), birth year (continuous), and the first five principal components (continuous).

^f^Adjusted model in BMI change were adjusted for age (continuous), age-squared (continuous), sex (male or female), birth year (continuous), the first five principal components (continuous), and BMI at 20 (continuous).

**Table 4.** Seven identified SNPs associated with the current BMI adjusted for total energy intake

SNP	Candidate gene(s)	Current BMI			

		Adjusted model^a^		Adjusted model + total energy^d^/weight^e^	

		β^b^	*P*-value^c^	β^b^	*P*-value^c^
rs4409766	*BORCS7-ASMT*	0.181	**4.96E-05**	0.036	2.93E-01
rs11030100	*BDNF-AS*	−0.199	**1.68E-06**	−0.082	8.95E-03
rs6265	*BDNF*	−0.203	**1.00E-06**	−0.071	2.26E-02
rs11030104	*BDNF-AS*	−0.203	**1.01E-06**	−0.067	3.18E-02
rs1421085	*FTO*	0.335	**1.24E-10**	0.174	**8.02E-06**
rs11642015	*FTO*	0.334	**7.36E-11**	0.173	**8.41E-06**
rs1558902	*FTO*	0.331	**8.65E-11**	0.174	**7.69E-06**

BMI, body mass index; SNP, single nucleotide polymorphism.

^a^Adjusted models were adjusted for age (continuous), age-squared (continuous), sex (male or female), birth year (continuous), and the top 5 principal components (continuous).

^b^Alternative alleles were treated as effect alleles.

^c^Significant *P*-values (*P* ≤ 1.77E-04) are shown in bold.

^d^Total energy was estimated using a self-reported food frequency questionnaire.

^e^Weight indicates self-reported current weight.

To further examine potential heterogeneity of susceptibility loci across multiple BMI-related traits, we estimated the heritability of each BMI phenotype (Table 5). In all participants, the heritability was 27.2% in current BMI, and 21.6% in BMI at 20. Of note, the estimated heritability was quite different between sex especially in current BMI (male: 42.2% vs female: 25.9%), and the proportion of effects of genetic factor in male were clearly changed from BMI at 20 to current BMI, but more stable in females than males. In addition, the pair-wise genetic correlation analysis among three BMI-related traits showed that current BMI and BMI change had higher correlation (75.7%) in contrast to BMI at 20 and BMI change (13.0%) (Table 5).

**Table 5.** Heritability of each phenotype and genetic correlation between phenotypes

(i) Heritability									

	All participants			Male			Female		
	(*n* = 11,586)			(*n* = 5,169)			(*n* = 6,417)		

	Heritability	SE	*P*-value^e^	Heritability	SE	*P*-value^e^	Heritability	SE	*P*-value^e^
Current BMI^a^	0.272	0.030	0.00E+00	0.422	0.065	1.64E-11	0.259	0.053	4.99E-07
BMI at 20^b^	0.216	0.029	4.22E-15	0.254	0.064	2.35E-05	0.322	0.053	1.99E-10
BMI change^c^	0.158	0.030	3.34E-08	0.239	0.066	1.90E-04	0.166	0.052	1.05E-03
BMI change (without adjustment for BMI at 20)	0.116	0.029	4.64E-05	0.131	0.065	2.95E-02	0.163	0.052	1.19E-03

(ii) Genetic correlation (*n* = 11,586)				

	Genetic correlation (rG)	SE	*P*-value^e^ (one-tailed, rG = 1)	*P*-value^e^ (one-tailed, rG = 0)
Current BMI^a^/BMI at 20^b^	0.753	0.063	9.72E-05	3.33E-16
Current BMI^a^/BMI change^d^	0.757	0.063	3.07E-03	5.89E-08
BMI at 20^b^/BMI change^d^	0.130	0.148	5.84E-04	1.77E-01

BMI, body mass index; SE, standard error.

^a^The covariates in current BMI were age (continuous), age-squared (continuous), birth year (continuous), sex (male or female), and first five principal components (continuous).

^b^The covariates in BMI at 20 were adjusted for sex (male or female), birth year (continuous), and the first five principal components (continuous).

^c^The covariates in BMI change were age (continuous), age-squared (continuous), sex (male or female), birth year (continuous), the first five principal components (continuous), and BMI at 20 (continuous).

^d^The covariates in BMI change were age (continuous), age-squared (continuous), sex (male or female), birth year (continuous), and the first five principal components (continuous).

^e^Significant *P*-values were defined as *P* ≤ 0.05.

## DISCUSSION

Our cross-sectional study within a prospective cohort study in a Japanese population showed that there was a significant association between BMI traits and polymorphisms in or near *FTO*, *BDNF*, *TMEM18*, *HS6ST3*, and *BORCS7*. All of these significant loci or genes have previously been reported in other ethnicities and are well-known BMI-associated polymorphisms.^37^^–^^47^ Our findings confirm the robustness of these associations in the Japanese population. Among the three BMI-related phenotypes examined in this study (current BMI, BMI at 20, and BMI change), the trend of association was similar in current BMI and BMI change, but differed from that in the BMI at 20. Interestingly, while *FTO* variants were associated with all BMI traits, *TMEM18* and *HS6ST3* were only associated with BMI at 20. Our results indicate that the effects of individual polymorphisms on BMI may vary across the life course.

A few studies have identified heterogeneities in genetic effects across age using both cross-sectional and longitudinal designs. Given that *FTO* loci reportedly explain the majority of the inter-individual variance among previously confirmed BMI-related loci,^41^ several studies have focused on *FTO* variants to explore the potential differential effects across the life course.^48^^–^^51^ One study revealed that the *FTO* variant identified by a GWAS in adults also showed a significant association in children and adolescents.^48^ This is consistent with our findings, suggesting that the *FTO* loci affect BMI across the life course. Another study showed that while there are consistent trends in the association of BMI with *FTO* loci, the effect size varies across life epoch.^50^ This study reported that among the four distinct phases of adulthood (18 to over 70 years), the strongest association between *FTO* loci and BMI was observed in young adults (18–25 years) relative to each life epoch.^50^ Similarly, another cohort study that conducted a longitudinal observation reported that the association between *FTO* polymorphisms and BMI strengthened with age, peaking at age 20 years, before weakening in later adult years.^49^ While the available evidence on *TMEM18* loci is scarcer than that for *FTO* loci, several GWASs have reported an association between *TMEM18* loci and BMI in both adults and children.^14^^,^^38^^,^^43^^,^^52^ Moreover, one study showed that there was no *TMEM18* loci heterogeneity across the life course.^50^ In contrast, we found that *TMEM18* variants were only associated with BMI at 20. However, the variants reported in previous life course studies differed from the loci examined in our study, which may explain the discordance in results. Importantly, our findings may suggest that the effect of some genes on BMI can differ across age while of other genes can be kept across age. In addition, each gene variant may exhibit different patterns of association with BMI, such that while one gene may have congenital effects, another may exhibit effects through an acquired pathway. Our findings in the current BMI and BMI change were similar, which supports the speculation that these associated significant genes affect BMI through an acquired pathway.

In particular, we think that dietary intake, for which habits are acquired after birth, may be the most important acquired pathway for obesity. Many obesity-related genes are expressed or are known to act in the central nervous system, especially in the hypothalamus, and the association between the expression of these genes and BMI may explain increases or decreases in appetite, such as via regulation of the feeding center in the hypothalamus.^38^^,^^41^ However, even after adjusting for total energy intake, *FTO* loci remained significantly associated with current BMI and BMI change. This may be because weight regulation by genetic factors is not simply dependent on feeding behavior, but also on other metabolic pathways and factors. For example, *FTO* loci are associated with an adipocyte thermogenesis regulation pathway involving *IRX3* and *IRX5*,^53^ and *BDNF* is associated with physical activity.^54^^,^^55^ Given that the mechanism governing the effects of genes on obesity remains poorly understood, further studies are required to clarify the genetic associations with obesity.

Results of heritability and genetic correlation also supported our speculation that genes which affect current BMI and BMI at 20 are heterogeneous. The estimated heritability in current BMI was higher than in BMI at 20 especially in males, and genetic correlation between BMI change and BMI at 20 were only 13% in contrast to 75% of consistency in current BMI and BMI change. These results can make it reasonable to suppose that the degree of dependent on BMI by genetic factors are different by age and genetic effects vary across life course.

This study has limitations that warrant mention. First, the statistical power may have been limited because of the sample size and strict application of the Bonferroni threshold. This may explain the few significant variants obtained in this study compared to a previous Japanese study.^15^ However, the variants that we identified as being significant under these conditions are likely strongly associated with BMI in the Japanese population and may code potential target genes for the development of measures against obesity in the future. Second, because the analysis was restricted to individuals of Japanese ancestry, our results may not be generalizable to other ethnic populations. Third, although we assumed that BMI is changed monotonically through the life course, the actual change may vary depending on the time. As a result, genetic effects over a period of time may be underestimated, whereas those in another period overestimated.

In conclusion, previously reported BMI-associated genes discovered in European populations were also identified in a Japanese population. Additionally, some of the significant variants identified in this study were associated with BMI at 20, but not with current BMI or BMI change. This indicates that the genetic effects on BMI may vary across the life course via different pathways. Further studies are needed to identify changes in effect sizes across the life course and the mechanisms of genetic effects on obesity in various ethnicities.

## APPENDIX A. SUPPLEMENTARY DATA

eTable 1. List of all SNPs examined

eTable 2. List of SNPs excluded from the initial candidate variants for analysis

eTable 3. Sex-stratified analysis of the 11 significant SNPs

eTable 4. Analysis of 11 significant SNPs excluding DM population in current BMI

eFigure 1. Consort diagram of the eligibility of subjects after merging questionnaire and genotype data. FFQ, food frequency questionnaire.

eFigure 2. Consort diagram of the selection of candidate loci. BBJ, BioBank Japan study^15^; BMI, body mass index; MAF, minor allele frequency; SNPs, single nucleotide polymorphisms.
