# Supplementary material for: Differential Effect of Polymorphisms on Body Mass Index Across the Life Course of Japanese: The Japan Multi-Institutional Collaborative Cohort Study
Source: J Epidemiol. 2021 Mar 5;31(3):172–9. doi: 10.2188/jea.JE20190296 (PMC7878711; doi:10.2188/jea.JE20190296)
Supplement: Supplementary file 1 [file je-31-172-s001.pdf]

eTable 1. List of all SNPs examined

SNP	Current BMI[a]	BMI at 20[b]	BMI change[c]	Chr.[d]	Position (bp)[d]	Candidate gene(s)	Current BMI			BMI at 20			BMI change		
							Adjusted[g]			Adjusted[g]			Adjusted[g]		
							β[e]	SE	*P* -value[f]	β[e]	SE	*P* -value[f]	β[e]	SE	*P* -value[f]
rs12044597	+	-	-	1	1708801	*NADK*	0.015	0.041	0.718	-0.005	0.031	0.863	0.000	0.001	0.849
rs2076463	+	-	-	1	27971092	*FGR, IFI6*	0.032	0.045	0.477	-0.008	0.034	0.808	0.001	0.001	0.566
rs657452	+	-	-	1	49589847	*AGBL4*	0.007	0.042	0.875	0.016	0.032	0.614	0.000	0.001	0.980
rs3101336	+	+	-	1	72751185	*NEGR1, LINC01360*	0.050	0.075	0.510	-0.029	0.057	0.606	0.003	0.002	0.183
rs12566985	+	+	-	1	75002193	*FPGT-TNNI3K, TNNI3K*	-0.159	0.074	0.030	-0.133	0.056	0.016	-0.003	0.002	0.196
rs12041852	-	+	-	1	75003500	*FPGT-TNNI3K, TNNI3K*	-0.159	0.074	0.030	-0.133	0.056	0.016	-0.003	0.002	0.196
rs11165643	+	-	-	1	96924097	*LINC01787, PTBP2*	-0.019	0.046	0.677	-0.019	0.035	0.582	0.000	0.001	0.878
rs11185092	+	-	-	1	107886278	*NTNG1*	0.059	0.055	0.282	0.048	0.041	0.245	0.001	0.002	0.737
rs10923724	+	-	-	1	119546842	*LOC105378933, WARS2*	0.068	0.042	0.103	0.068	0.032	0.030	0.001	0.001	0.627
rs860295	+	-	-	1	155767708	*GON4L*	0.080	0.047	0.089	0.009	0.036	0.811	0.002	0.001	0.102
rs1749405	+	-	-	1	155860091	*SYT11, RIT1*	0.069	0.050	0.166	0.017	0.038	0.661	0.002	0.001	0.248
rs347313	-	-	+	1	162304276	*NOS1AP*	-0.080	0.051	0.117	-0.070	0.039	0.070	0.000	0.002	0.874
rs633715	+	-	-	1	177852580	*LINC01741, SEC16B*	0.064	0.049	0.190	0.087	0.037	0.019	0.000	0.001	0.768
rs543874	+	+	-	1	177920345	*LINC01741, SEC16B*	0.058	0.049	0.240	0.081	0.037	0.029	0.000	0.001	0.842
rs10920678	+	-	-	1	190239907	*BRINP3*	0.016	0.043	0.714	0.039	0.033	0.225	0.000	0.001	0.991
rs491055	+	-	-	1	190308834	*BRINP3*	0.024	0.043	0.574	0.054	0.032	0.092	0.000	0.001	0.970
rs10754220	+	-	-	1	197244290	*CRB1*	-0.114	0.054	0.035	0.011	0.041	0.794	-0.003	0.002	0.067
rs2820315	+	-	-	1	201872264	*LMOD1*	-0.110	0.069	0.112	-0.083	0.052	0.112	-0.001	0.002	0.509
rs823114	+	-	-	1	205719532	*NUCKS1*	0.071	0.041	0.080	0.042	0.031	0.168	0.002	0.001	0.135
rs9786986	+	-	-	1	235656632	*B3GALNT2*	0.036	0.041	0.381	0.033	0.031	0.281	0.001	0.001	0.672
rs939584	+	-	-	2	621558	*LOC105373352, TMEM18*	-0.220	0.067	0.001	-0.217	0.051	0.000	-0.003	0.002	0.152
rs13021737	+	+	-	2	632348	*LOC105373352, TMEM18*	-0.214	0.067	0.001	-0.208	0.051	0.000	-0.003	0.002	0.177
rs4854349	-	+	-	2	647861	*LOC105373352, TMEM18*	-0.191	0.067	0.004	-0.190	0.051	0.000	-0.002	0.002	0.230
rs111612372	+	-	-	2	20433218	*SDC1, PUM2*	0.059	0.051	0.247	-0.014	0.039	0.718	0.002	0.002	0.188
rs11676272	-	+	-	2	25141538	*ADCY3*	0.136	0.040	0.001	0.078	0.030	0.011	0.003	0.001	0.015
rs10182181	+	+	-	2	25150296	*ADCY3, DNAJC27*	0.137	0.040	0.001	0.076	0.030	0.013	0.003	0.001	0.014
rs713586	+	-	-	2	25158008	*ADCY3, DNAJC27*	-0.118	0.040	0.004	-0.058	0.030	0.056	-0.003	0.001	0.017
rs11126666	+	-	-	2	26928811	*KCNK3*	-0.025	0.043	0.567	-0.057	0.033	0.082	-0.001	0.001	0.581
rs6734118	+	-	-	2	37559355	*PRKD3, QPCT*	-0.031	0.041	0.446	-0.011	0.031	0.730	-0.001	0.001	0.677
rs77489951	+	-	-	2	38750287	*LOC101929596, HNRNPLL*	0.048	0.086	0.572	0.093	0.065	0.151	0.001	0.003	0.726
rs4596023	+	-	-	2	48955683	*LHCGR, STON1-GTF2A1L*	-0.074	0.041	0.069	0.001	0.031	0.971	-0.002	0.001	0.053
rs10174398	+	-	-	2	51195601	*NRXN1*	0.033	0.041	0.426	0.061	0.031	0.051	0.000	0.001	0.896
rs10197655	+	-	-	2	58791420	*LINC01122*	0.086	0.041	0.037	0.078	0.031	0.013	0.001	0.001	0.277
rs4671328	+	-	-	2	58935282	*LINC01122*	0.038	0.042	0.363	0.088	0.032	0.005	0.000	0.001	0.721
rs11688816	+	-	-	2	63053048	*EHBP1*	0.050	0.048	0.300	0.028	0.036	0.443	0.001	0.001	0.679
rs12617004	+	-	-	2	142615136	*LRP1B*	0.030	0.042	0.464	-0.025	0.031	0.427	0.000	0.001	0.756
rs2390669	+	-	-	2	169091942	*STK39*	0.015	0.048	0.762	0.026	0.037	0.472	0.000	0.001	0.978
rs6433857	+	-	-	2	181517996	*CWC22, SCHLAP1*	-0.034	0.043	0.426	0.020	0.033	0.551	-0.002	0.001	0.200
rs1528435	+	-	-	2	181550962	*CWC22, SCHLAP1*	-0.035	0.044	0.429	0.008	0.033	0.821	-0.002	0.001	0.248
rs7569376	+	-	-	2	205385322	*ICOS, PARD3B*	-0.019	0.041	0.637	-0.003	0.031	0.911	0.000	0.001	0.703
rs972540	+	-	-	2	207244783	*ZDBF2, ADAM23*	0.056	0.054	0.303	-0.002	0.041	0.964	0.002	0.002	0.170
rs11692326	+	-	-	2	208263279	*LINC01802, CREB1*	-0.027	0.056	0.631	-0.027	0.042	0.518	-0.001	0.002	0.389
rs7565158	-	-	+	2	213593970	*ERBB4, LINC01878*	0.023	0.041	0.584	0.016	0.031	0.596	0.000	0.001	0.963
rs4072096	+	-	-	2	227036603	*LOC646736*	-0.064	0.041	0.123	0.006	0.031	0.837	-0.001	0.001	0.248
rs2574704	+	-	-	3	11655381	*VGLL4*	-0.008	0.043	0.843	-0.063	0.032	0.050	0.001	0.001	0.351
rs10510554	+	-	-	3	25099776	*RARB*	-0.005	0.041	0.904	-0.043	0.031	0.170	0.001	0.001	0.526
rs8192473	+	-	-	3	42299399	*CCK*	-0.024	0.069	0.725	-0.086	0.052	0.100	0.001	0.002	0.570
rs10460960	+	-	-	3	42308735	*CCK, LYZL4*	-0.038	0.045	0.398	-0.023	0.034	0.497	-0.001	0.001	0.697
rs7613875	+	-	-	3	49971514	*MON1A, RBM6*	0.043	0.055	0.437	-0.025	0.042	0.550	0.002	0.002	0.128
rs11130319	+	-	-	3	52755592	*NEK4*	-0.066	0.041	0.105	-0.053	0.031	0.085	-0.001	0.001	0.559
rs2240920	+	-	-	3	52831009	*ITIH3*	0.051	0.041	0.207	0.023	0.031	0.456	0.001	0.001	0.493
rs2365389	+	-	-	3	61236462	*FHIT*	0.011	0.071	0.881	0.044	0.054	0.412	-0.002	0.002	0.438
rs12495178	+	-	-	3	85886077	*CADM2*	-0.050	0.044	0.251	-0.014	0.033	0.669	0.000	0.001	0.763
rs2868975	+	-	-	3	116935323	*LINC00901, LINC02024*	-0.065	0.047	0.173	-0.042	0.036	0.237	-0.001	0.001	0.357
rs1225051	+	-	-	3	131645972	*CPNE4*	-0.061	0.041	0.135	-0.048	0.031	0.121	-0.001	0.001	0.530
rs7621025	+	-	-	3	136272246	*STAG1*	-0.035	0.052	0.506	0.001	0.040	0.978	-0.001	0.002	0.676
rs16851483	+	-	-	3	141275436	*RASA2*	0.057	0.045	0.207	0.030	0.034	0.375	0.001	0.001	0.277
rs188981	-	-	+	3	151854115	*ADACL2-AS1, LOC101928166*	-0.033	0.045	0.459	-0.058	0.034	0.087	-0.001	0.001	0.602
rs4686392	+	-	-	3	185524081	*IGF2BP2*	-0.046	0.043	0.283	0.025	0.033	0.444	-0.001	0.001	0.345
rs3774854	-	-	+	4	5711368	*EVC2*	0.144	0.045	0.001	0.093	0.034	0.006	0.002	0.001	0.140
rs1996023	+	-	-	4	45164637	*GNPDA2, GABRG1*	0.137	0.045	0.003	0.070	0.034	0.040	0.002	0.001	0.079
rs13130484	-	+	-	4	45175691	*GNPDA2, GABRG1*	-0.012	0.041	0.779	0.022	0.031	0.482	-0.001	0.001	0.220
rs10938397	+	+	-	4	45182527	*GNPDA2, GABRG1*	0.147	0.045	0.001	0.094	0.034	0.006	0.002	0.001	0.129
rs1229984	+	-	-	4	100239319	*ADH1B*	0.030	0.046	0.516	0.030	0.035	0.385	0.000	0.001	0.820
rs4834272	+	-	-	4	113313986	*ALPK1*	-0.073	0.041	0.072	0.011	0.031	0.721	-0.002	0.001	0.160
rs16869212	-	-	+	5	16882090	*MYO10*	0.003	0.042	0.934	0.048	0.032	0.131	0.000	0.001	0.928
rs7720894	+	-	-	5	60733933	*ZSWIM6*	-0.031	0.044	0.481	-0.024	0.033	0.476	-0.001	0.001	0.277
rs1035491	+	-	-	5	63962177	*RGS7BP, FAM159B*	-0.002	0.046	0.963	0.004	0.035	0.909	0.001	0.001	0.548
rs6881648	+	-	-	5	74991849	*POC5*	0.043	0.041	0.293	0.009	0.031	0.782	0.001	0.001	0.237
rs2112347	+	-	-	5	75015242	*POC5, SV2C*	0.044	0.041	0.284	0.009	0.031	0.779	0.001	0.001	0.255
rs1846974	+	-	-	5	87969927	*LINC00461*	0.044	0.041	0.283	-0.008	0.031	0.791	0.002	0.001	0.104
rs16903285	+	-	-	5	87978252	*LINC00461, MEF2C-AS2*	0.026	0.041	0.523	-0.020	0.031	0.523	0.002	0.001	0.136
rs10062657	+	-	-	5	95867908	*LOC101929710*	0.138	0.042	0.001	0.084	0.032	0.008	0.002	0.001	0.191
rs1582931	+	-	-	5	122657199	*PRDM6, CEP120*	0.099	0.041	0.015	0.090	0.031	0.003	0.001	0.001	0.423
rs4357030	+	-	-	5	124316031	*ZNF608, LOC101927421*	-0.053	0.041	0.195	0.019	0.031	0.542	-0.002	0.001	0.091
rs6864049	+	-	-	5	124330522	*ZNF608, LOC101927421*	-0.013	0.044	0.776	0.022	0.033	0.507	0.000	0.001	0.812
rs329120	+	-	-	5	133861756	*JADE2*	0.037	0.042	0.377	-0.061	0.032	0.055	0.003	0.001	0.023
rs329122	+	-	-	5	133864599	*JADE2*	0.037	0.042	0.377	-0.061	0.032	0.055	0.003	0.001	0.023
rs72812846	+	-	-	5	173377636	*CPEB4*	0.001	0.069	0.990	0.015	0.052	0.770	-0.001	0.002	0.708
rs2228213	+	-	-	6	12124855	*HIVEP1*	-0.037	0.046	0.413	-0.045	0.034	0.193	0.000	0.001	0.713
rs35261542	+	-	-	6	20675792	*CDKAL1*	-0.040	0.042	0.338	-0.033	0.031	0.296	0.000	0.001	0.696
rs9368222	+	-	-	6	20686996	*CDKAL1*	-0.042	0.042	0.310	-0.030	0.031	0.333	-0.001	0.001	0.636
rs6913361	+	-	-	6	34179390	*GRM4, HMGA1*	-0.032	0.061	0.594	0.053	0.046	0.243	-0.002	0.002	0.367
rs10947487	+	-	-	6	34189612	*GRM4, HMGA1*	-0.039	0.061	0.519	0.053	0.046	0.244	-0.002	0.002	0.314
rs2033529	+	-	-	6	40348653	*TDRG1, LRFN2*	-0.005	0.045	0.917	0.010	0.034	0.776	0.000	0.001	0.999
rs2206271	+	-	-	6	50786008	*TFAP2B*	0.100	0.043	0.019	0.052	0.032	0.108	0.001	0.001	0.377
rs2206277	+	-	-	6	50798526	*TFAP2B*	0.092	0.044	0.037	0.037	0.033	0.270	0.001	0.001	0.259
rs987237	-	+	-	6	50803050	*TFAP2B*	0.056	0.050	0.260	0.021	0.038	0.583	0.001	0.001	0.650
rs2207139	-	+	-	6	50845490	*TFAP2B, PKHD1*	0.026	0.047	0.584	-0.006	0.036	0.858	0.001	0.001	0.700
rs9346455	-	-	+	6	71991816	*B3GAT2, OGFRL1*	-0.018	0.054	0.746	0.056	0.041	0.172	-0.002	0.002	0.301
rs947612	+	-	-	6	73738661	*KCNQ5*	0.013	0.051	0.796	0.013	0.039	0.746	0.000	0.002	0.831
rs3800229	+	-	-	6	108996963	*FOXO3*	-0.180	0.050	0.000	-0.056	0.037	0.133	-0.005	0.001	0.002
rs7756389	+	-	-	6	114064674	*LOC101927686, MARCKS*	0.022	0.041	0.586	0.032	0.031	0.309	0.000	0.001	0.758
rs2357760	+	-	-	6	120213880	*LOC105377975, MIR3144*	0.018	0.044	0.685	-0.009	0.033	0.789	0.000	0.001	0.722
rs2185027	+	-	-	6	153381622	*RGS17*	-0.054	0.041	0.193	-0.026	0.031	0.396	-0.001	0.001	0.223
rs9397585	+	-	-	6	153396875	*RGS17*	-0.084	0.043	0.050	-0.019	0.032	0.556	-0.002	0.001	0.086
rs487152	+	-	-	6	160774486	*SLC22A3*	0.065	0.045	0.155	0.028	0.034	0.408	0.001	0.001	0.531
rs10248288	+	-	-	7	27251776	*HOTTIP, EVX1-AS*	0.055	0.041	0.176	0.048	0.031	0.119	0.002	0.001	0.194
rs7779181	+	-	-	7	32345283	*PDE1C*	0.061	0.048	0.210	0.068	0.037	0.063	0.000	0.001	0.882
rs2908296	+	-	-	7	44193462	*GCK*	-0.029	0.044	0.521	-0.019	0.034	0.577	0.000	0.001	0.983
rs10269783	+	-	-	7	49616203	*CDC14C, VWC2*	-0.039	0.041	0.338	0.012	0.031	0.689	-0.002	0.001	0.123
rs6947395	+	-	-	7	69406661	*AUTS2*	-0.073	0.052	0.158	-0.083	0.039	0.035	-0.001	0.002	0.579
rs1949804	+	-	-	7	69768826	*AUTS2*	-0.066	0.056	0.239	-0.087	0.042	0.039	-0.001	0.002	0.760
rs3930017	+	-	-	7	76720582	*FAM185BP*	-0.026	0.056	0.635	-0.074	0.042	0.078	0.000	0.002	0.849
rs3779273	+	-	-	7	77828940	*MAGI2*	0.001	0.041	0.977	-0.062	0.031	0.044	0.002	0.001	0.105
rs2237579	+	-	-	7	93095867	*CALCR*	0.004	0.042	0.930	0.043	0.032	0.176	0.000	0.001	0.843
rs7786765	-	-	+	7	93199928	*CALCR*	0.036	0.046	0.440	0.008	0.035	0.825	0.001	0.001	0.657
rs634010	-	-	+	7	111698108	*DOCK4*	0.013	0.050	0.788	0.009	0.038	0.820	0.000	0.001	0.873
rs6950442	+	-	-	7	114412664	*FOXP2, MDFIC*	0.072	0.049	0.146	0.067	0.037	0.071	0.001	0.001	0.421
rs143665886	+	-	-	7	115368366	*SNORA25B, TFEC*	-0.007	0.041	0.872	0.062	0.031	0.047	-0.001	0.001	0.337
rs1809626	+	-	-	7	135686420	*MTPN, CHRM2*	-0.023	0.048	0.638	0.042	0.037	0.250	-0.001	0.001	0.524
rs6960319	-	-	+	7	147258631	*CNTNAP2*	-0.077	0.047	0.100	0.007	0.036	0.842	-0.004	0.001	0.005
rs3758141	-	-	+	8	18727949	*PSD3*	0.055	0.040	0.174	0.051	0.031	0.095	0.001	0.001	0.436
rs13253111	-	+	-	8	28061974	*ELP3, PNOC*	0.076	0.044	0.081	0.080	0.033	0.016	0.000	0.001	0.968
rs12156392	+	-	-	8	28140103	*ELP3, PNOC*	0.025	0.045	0.578	-0.005	0.034	0.892	0.001	0.001	0.424
rs10091344	+	-	-	8	34132075	*DUSP26, LINC01288*	0.064	0.041	0.122	0.005	0.031	0.876	0.002	0.001	0.131
rs77636220	+	-	-	8	64552779	*LOC102724612, LINC01289*	0.035	0.051	0.492	0.045	0.038	0.242	-0.001	0.001	0.563
rs11787111	+	-	-	8	65192451	*LOC102724623*	0.082	0.049	0.091	0.051	0.037	0.164	0.001	0.001	0.455
rs28857569	+	-	-	8	76697034	*HNF4G, LINC01111*	0.096	0.044	0.028	0.061	0.033	0.063	0.002	0.001	0.156
rs17405819	+	-	-	8	76806584	*HNF4G, LINC01111*	-0.095	0.042	0.023	-0.053	0.032	0.093	-0.002	0.001	0.077
rs16907751	+	-	-	8	81375457	*MIR5708, ZBTB10*	-0.172	0.059	0.004	-0.071	0.045	0.112	-0.004	0.002	0.018
rs2033732	+	-	-	8	85079709	*LINC01419, RALYL*	-0.017	0.046	0.708	-0.018	0.035	0.595	0.000	0.001	0.765
rs4366055	+	-	-	8	95507328	*VIRMA*	-0.067	0.040	0.099	-0.028	0.031	0.360	-0.001	0.001	0.296
rs12680842	+	-	-	8	95582606	*VIRMA, LOC100288748*	-0.067	0.040	0.100	-0.040	0.031	0.191	-0.001	0.001	0.419
rs3802177	+	-	-	8	118185025	*SLC30A8*	0.006	0.041	0.876	-0.018	0.031	0.557	0.001	0.001	0.636
rs9650069	+	-	-	8	118204020	*SLC30A8, MED30*	0.014	0.041	0.729	-0.014	0.031	0.650	0.001	0.001	0.576
rs4740619	+	-	-	9	15634326	*CCDC171*	0.017	0.049	0.732	-0.068	0.037	0.067	0.002	0.001	0.289
rs7020996	+	-	-	9	22129579	*CDKN2B-AS1, DMRTA1*	0.035	0.041	0.402	0.027	0.031	0.380	0.001	0.001	0.526
rs10811661	+	-	-	9	22134094	*CDKN2B-AS1, DMRTA1*	0.041	0.041	0.321	0.030	0.031	0.326	0.001	0.001	0.572
rs10811901	+	-	-	9	23356935	*LINC01239, LOC101929563*	-0.045	0.049	0.351	-0.081	0.037	0.027	0.001	0.001	0.705
rs2183824	+	-	-	9	28412078	*LINGO2*	0.037	0.046	0.424	0.021	0.034	0.547	0.000	0.001	0.940
rs11140653	-	-	+	9	87152509	*SLC28A3, NTRK2*	-0.039	0.057	0.499	0.006	0.043	0.898	-0.002	0.002	0.374
rs10868215	+	-	-	9	87234111	*SLC28A3, NTRK2*	-0.086	0.044	0.048	-0.031	0.033	0.349	-0.003	0.001	0.014
rs1147199	+	-	-	9	87275895	*SLC28A3, NTRK2*	-0.065	0.045	0.152	0.002	0.034	0.948	-0.002	0.001	0.068
rs10820904	+	-	-	9	94568424	*ROR2*	-0.009	0.046	0.841	-0.031	0.035	0.380	0.000	0.001	0.950
rs12236219	+	-	-	9	97062981	*ZNF169*	-0.046	0.044	0.298	-0.050	0.033	0.137	0.000	0.001	0.882
rs3932549	+	-	-	9	97073588	*ZNF169, NUTM2F*	-0.038	0.044	0.386	-0.048	0.033	0.150	0.000	0.001	0.982
rs580809	+	-	-	9	101518442	*ANKS6*	-0.064	0.041	0.120	0.011	0.031	0.718	-0.002	0.001	0.059
rs7024334	+	-	-	9	109072075	*TMEM38B, MIR8081*	-0.067	0.042	0.106	0.003	0.031	0.930	-0.002	0.001	0.108
rs6477694	+	-	-	9	111932342	*FRRS1L, EPB41L4B*	-0.015	0.042	0.728	-0.059	0.032	0.065	0.001	0.001	0.438
rs1928295	+	-	-	9	120378483	*ASTN2, LOC101928797*	-0.040	0.041	0.328	-0.083	0.031	0.007	0.000	0.001	0.888
rs5015933	+	-	-	9	128137418	*GAPVD1, MAPKAP1*	0.054	0.041	0.184	0.054	0.031	0.083	0.001	0.001	0.475
rs2235056	+	-	-	9	129377235	*LMX1B*	0.016	0.041	0.689	-0.005	0.031	0.868	0.001	0.001	0.555
rs3829849	-	+	-	9	129390800	*LMX1B*	-0.045	0.044	0.306	0.003	0.033	0.938	-0.001	0.001	0.425
rs2270204	+	-	-	9	131042734	*SWI5*	-0.017	0.041	0.681	0.007	0.031	0.825	0.000	0.001	0.723
rs59192760	+	-	-	9	131158938	*MIR1268A*	0.012	0.049	0.803	-0.010	0.037	0.778	0.001	0.001	0.665
rs7042889	-	-	+	9	131795361	*SH3GLB2, MIGA2*	0.022	0.053	0.680	-0.023	0.040	0.565	0.002	0.002	0.148

rs10795945	+	-	-	10	12302607	*CDC123, CAMK1D*	-0.012	0.041	0.767	0.029	0.031	0.352	-0.001	0.001	0.531
rs11257655	+	-	-	10	12307894	*CDC123, CAMK1D*	-0.016	0.041	0.693	0.029	0.031	0.347	-0.001	0.001	0.468
rs1277723	+	-	-	10	18554623	*CACNB2*	-0.138	0.042	0.001	0.004	0.032	0.903	-0.005	0.001	0.000
rs7912454	+	-	-	10	18584792	*CACNB2*	-0.115	0.054	0.035	0.002	0.041	0.967	-0.004	0.002	0.022
rs11239187	-	-	+	10	45133277	*CXCL12, TMEM72-AS1*	0.080	0.053	0.133	-0.023	0.040	0.566	0.004	0.002	0.023
rs2163188	+	-	-	10	65314711	*REEP3*	0.019	0.044	0.668	-0.027	0.033	0.411	0.001	0.001	0.607
rs80117551	+	-	-	10	69834828	*HERC4*	-0.161	0.047	0.001	-0.069	0.035	0.051	-0.004	0.001	0.002
rs1832886	+	-	-	10	94477539	*HHEX, EXOC6*	-0.058	0.051	0.255	-0.015	0.039	0.699	-0.001	0.002	0.708
rs7923837	+	-	-	10	94481917	*HHEX, EXOC6*	-0.058	0.051	0.256	-0.016	0.039	0.674	-0.001	0.002	0.717
rs12569457	+	-	-	10	99096676	*FRAT2, RRP12*	-0.016	0.052	0.762	-0.010	0.039	0.796	0.000	0.002	0.836
rs17094222	+	-	-	10	102395440	*HIF1AN, PAX2*	0.029	0.043	0.500	0.064	0.033	0.051	0.000	0.001	0.861
rs2495707	+	-	-	10	102425949	*HIF1AN, PAX2*	-0.020	0.041	0.617	-0.056	0.031	0.067	0.001	0.001	0.471
rs4409766	+	-	-	10	104616663	*BORCS7-ASMT*	0.181	0.045	0.000	0.036	0.034	0.293	0.005	0.001	0.000
rs1907240	+	-	-	10	122897959	*WDR11, FGFR2*	0.057	0.044	0.197	0.059	0.033	0.076	0.000	0.001	0.737
rs2257129	+	-	-	10	122898697	*WDR11, FGFR2*	0.055	0.044	0.214	0.058	0.033	0.081	0.000	0.001	0.762
rs7090367	+	-	-	10	125234492	*BUB3, GPR26*	-0.056	0.042	0.183	-0.041	0.032	0.204	-0.001	0.001	0.541
rs1568079	+	-	-	10	125251751	*BUB3, GPR26*	-0.057	0.043	0.182	-0.042	0.032	0.187	-0.001	0.001	0.513
rs60808706	+	-	-	11	2857233	*KCNQ1*	0.088	0.041	0.034	0.067	0.031	0.032	0.001	0.001	0.253
rs2237897	+	-	-	11	2858546	*KCNQ1*	0.109	0.042	0.009	0.080	0.032	0.012	0.002	0.001	0.135
rs16937956	+	-	-	11	8404501	*LMO1, STK33*	0.089	0.042	0.032	0.036	0.031	0.246	0.003	0.001	0.040
rs10840099	+	-	-	11	8667680	*TRIM66*	0.123	0.042	0.004	0.092	0.032	0.004	0.003	0.001	0.034
rs5215	+	-	-	11	17408630	*KCNJ11*	-0.117	0.042	0.006	-0.028	0.032	0.381	-0.003	0.001	0.006
rs11030100	+	-	-	11	27677586	*BDNF-AS*	-0.199	0.042	0.000	-0.082	0.031	0.009	-0.005	0.001	0.000
rs6265	+	-	-	11	27679916	*BDNF*	-0.203	0.041	0.000	-0.071	0.031	0.023	-0.005	0.001	0.000
rs11030104	-	+	-	11	27684517	*BDNF-AS*	-0.203	0.042	0.000	-0.067	0.031	0.032	-0.005	0.001	0.000
rs3026401	+	-	-	11	31807524	*PAX6*	-0.015	0.041	0.713	0.023	0.031	0.465	-0.001	0.001	0.323
rs3817334	+	-	-	11	47650993	*MTCH2*	-0.025	0.043	0.571	0.008	0.033	0.804	-0.002	0.001	0.178
rs11602339	+	-	-	11	47761471	*FNBP4*	-0.015	0.043	0.728	0.017	0.033	0.614	-0.002	0.001	0.212
rs506338	+	-	-	11	64440920	*NRXN2*	0.023	0.054	0.674	-0.003	0.041	0.941	0.000	0.002	0.963
rs7123876	+	-	-	11	72444583	*ARAP1*	0.030	0.048	0.534	0.023	0.036	0.515	0.001	0.001	0.511
rs10899469	+	-	-	11	78018313	*GAB2*	-0.028	0.041	0.496	-0.052	0.031	0.095	-0.001	0.001	0.585
rs55633855	+	-	-	11	88134563	*CTSC, GRM5-AS1*	-0.006	0.061	0.918	-0.050	0.046	0.272	0.001	0.002	0.443
rs1048932	+	-	-	11	115044850	*CADM1*	0.079	0.041	0.054	0.053	0.031	0.090	0.002	0.001	0.190
rs10772983	+	-	-	12	17141582	*SKP1P2*	0.065	0.044	0.138	0.053	0.033	0.111	0.001	0.001	0.692
rs10841048	-	-	+	12	18779259	*PIK3C2G*	0.000	0.043	0.991	0.015	0.033	0.657	0.000	0.001	0.990
rs80234489	+	-	-	12	31441179	*FAM60A*	-0.103	0.051	0.043	-0.069	0.039	0.073	-0.003	0.002	0.080
rs4346053	-	-	+	12	33115820	*PKP2, SYT10*	0.056	0.045	0.222	0.043	0.034	0.213	0.001	0.001	0.475
rs7138803	+	+	-	12	50247468	*BCDIN3D, FAIM2*	0.000	0.043	0.991	0.015	0.033	0.657	0.000	0.001	0.990
rs3205718	+	-	-	12	50261809	*FAIM2*	0.058	0.045	0.198	0.044	0.034	0.195	0.001	0.001	0.445
rs7132908	-	+	-	12	50263148	*FAIM2*	-0.019	0.043	0.666	0.003	0.033	0.925	0.000	0.001	0.758
rs77511173	+	-	-	12	53883537	*MAP3K12*	-0.083	0.066	0.208	-0.061	0.050	0.218	-0.001	0.002	0.464
rs11105839	+	-	-	12	91237920	*LINC02392, LINC00615*	-0.029	0.041	0.478	0.031	0.031	0.318	-0.002	0.001	0.130
rs7305242	+	-	-	12	112256762	*ALDH2, MAPKAPK5-AS1*	0.065	0.043	0.130	0.056	0.032	0.081	0.001	0.001	0.364
rs2301712	+	-	-	12	112641377	*HECTD4*	0.056	0.043	0.191	0.049	0.032	0.132	0.001	0.001	0.446
rs10507285	-	-	+	12	118295924	*KSR2*	0.106	0.060	0.078	0.032	0.046	0.477	0.003	0.002	0.151
rs4319547	+	-	-	12	123079035	*KNTC1*	-0.063	0.058	0.274	-0.039	0.044	0.370	-0.001	0.002	0.437
rs1006353	+	-	-	13	28047269	*MTIF3, LNX2*	-0.020	0.042	0.635	0.001	0.032	0.965	-0.001	0.001	0.512
rs1751857	-	-	+	13	40969438	*LINC00598*	-0.027	0.054	0.616	-0.020	0.041	0.623	0.000	0.002	0.998
rs12429545	+	+	-	13	54102206	*LINC01065, LINC00558*	0.084	0.049	0.085	0.045	0.037	0.220	0.002	0.001	0.230
rs9568867	+	-	-	13	54107352	*LINC01065, LINC00558*	0.084	0.049	0.086	0.045	0.037	0.219	0.002	0.001	0.234
rs2321882	+	-	-	13	59451989	*LINC00374, DIAPH3*	0.001	0.064	0.987	0.069	0.049	0.155	-0.002	0.002	0.412
rs9540493	+	-	-	13	66205704	*LINC00355, LINC01052*	0.006	0.049	0.903	0.022	0.037	0.548	0.000	0.001	0.839
rs1927790	+	-	-	13	96922191	*HS6ST3*	0.057	0.041	0.164	0.119	0.031	0.000	0.000	0.001	0.834
rs10132280	+	-	-	14	25928179	*STXBP6, NOVA1*	0.054	0.079	0.499	0.070	0.060	0.245	0.000	0.002	0.931
rs12895330	+	-	-	14	33305343	*AKAP6, NPAS3*	0.131	0.050	0.008	0.084	0.038	0.025	0.003	0.001	0.060
rs712419	-	-	+	14	45203476	*LOC105370473, LINC02302*	-0.055	0.056	0.328	-0.005	0.042	0.912	-0.002	0.002	0.199
rs75766425	+	-	-	14	52511911	*NID2*	0.189	0.061	0.002	0.039	0.046	0.399	0.005	0.002	0.004
rs7141420	+	-	-	14	79899454	*NRXN3*	0.061	0.043	0.154	0.031	0.032	0.342	0.000	0.001	0.733
rs3783890	+	-	-	14	93790276	*BTBD7*	-0.032	0.043	0.455	-0.037	0.032	0.251	-0.001	0.001	0.595
rs729050	+	-	-	14	94109502	*UNC79*	0.036	0.042	0.387	0.006	0.032	0.852	0.001	0.001	0.247
rs709400	+	-	-	14	104149475	*KLC1*	-0.042	0.061	0.493	-0.034	0.046	0.456	-0.002	0.002	0.334
rs12440086	+	-	-	15	27038492	*GABRB3, GABRA5*	-0.083	0.042	0.047	-0.015	0.032	0.629	-0.002	0.001	0.194
rs1559677	+	-	-	15	47738063	*SEMA6D*	0.047	0.041	0.249	0.064	0.031	0.038	0.001	0.001	0.354
rs9888736	+	-	-	15	47940812	*SEMA6D*	-0.133	0.041	0.001	-0.100	0.031	0.001	-0.001	0.001	0.272
rs2593235	+	-	-	15	57541201	*TCF12*	-0.005	0.042	0.905	-0.050	0.031	0.108	0.001	0.001	0.575
rs17303831	+	-	-	15	62134332	*LINC02349, VPS13C*	-0.073	0.049	0.134	-0.021	0.037	0.569	-0.002	0.001	0.087
rs72749754	+	-	-	15	62319432	*VPS13C*	-0.091	0.048	0.059	-0.038	0.036	0.293	-0.003	0.001	0.065
rs4776970	+	-	-	15	68080886	*MAP2K5*	0.096	0.050	0.053	0.013	0.038	0.725	0.003	0.001	0.031
rs936674	-	-	+	15	93903597	*RGMA, LINC02207*	0.045	0.043	0.296	0.009	0.033	0.772	0.001	0.001	0.434
rs879620	+	-	-	16	4015729	*ADCY9*	0.145	0.043	0.001	0.080	0.033	0.014	0.003	0.001	0.012
rs2540034	+	-	-	16	4022694	*ADCY9*	0.126	0.044	0.004	0.069	0.033	0.036	0.003	0.001	0.030
rs9302817	+	-	-	16	6163936	*RBFOX1*	-0.091	0.043	0.033	-0.020	0.032	0.534	-0.002	0.001	0.136
rs7200543	+	-	-	16	15129970	*PDXDC1*	0.017	0.041	0.678	0.023	0.031	0.460	0.000	0.001	0.849
rs8046312	-	+	-	16	19979334	*GPRC5B, GPR139*	-0.035	0.044	0.430	-0.047	0.033	0.158	0.000	0.001	0.836
rs4238585	+	-	-	16	20255097	*GPR139, GP2*	-0.133	0.053	0.012	-0.117	0.040	0.003	-0.001	0.002	0.393
rs12597682	+	-	-	16	20258432	*GPR139, GP2*	-0.128	0.053	0.015	-0.117	0.040	0.003	-0.001	0.002	0.425
rs7195386	+	-	-	16	24578458	*RBBP6*	-0.017	0.041	0.681	-0.046	0.031	0.140	0.001	0.001	0.499
rs62034325	+	-	-	16	28538640	*IL27, NUPR1*	0.153	0.062	0.014	0.165	0.047	0.000	0.002	0.002	0.401
rs3888190	+	-	-	16	28889486	*ATP2A1, ATP2A1-AS1*	0.093	0.058	0.110	0.095	0.044	0.030	0.001	0.002	0.629
rs2080454	+	-	-	16	49062590	*MIR5095, CBLN1*	-0.008	0.041	0.841	-0.056	0.031	0.072	0.001	0.001	0.495
rs1564981	+	-	-	16	50986308	*LINC02168, LINC02127*	-0.068	0.041	0.097	-0.052	0.031	0.096	-0.001	0.001	0.226
rs17795934	+	-	-	16	51926509	*LINC01571, C16orf97*	-0.023	0.042	0.576	-0.017	0.031	0.590	0.000	0.001	0.840
rs1421085	-	+	-	16	53800954	*FTO*	0.331	0.051	0.000	0.174	0.039	0.000	0.006	0.002	0.000
rs11642015	+	-	-	16	53802494	*FTO*	0.335	0.051	0.000	0.173	0.039	0.000	0.007	0.002	0.000
rs1558902	+	-	+	16	53803574	*FTO*	0.334	0.051	0.000	0.174	0.039	0.000	0.007	0.002	0.000
rs2307022	+	-	-	16	68381978	*PRMT7*	0.093	0.063	0.138	0.063	0.047	0.183	0.001	0.002	0.589
rs8044868	-	-	+	16	72162102	*PMFBP1*	-0.001	0.042	0.983	0.044	0.031	0.164	-0.001	0.001	0.678
rs756717	+	-	-	16	72996162	*ZFHX3*	-0.062	0.044	0.161	-0.018	0.033	0.592	-0.002	0.001	0.211
rs4788694	+	-	-	16	73070083	*ZFHX3*	0.120	0.044	0.006	0.037	0.033	0.264	0.003	0.001	0.008
rs12926503	-	-	+	16	84031482	*NECAB2*	0.031	0.062	0.621	0.021	0.047	0.649	0.001	0.002	0.560
rs2281727	+	-	-	17	2117945	*SMG6*	0.068	0.050	0.172	0.009	0.038	0.808	0.002	0.001	0.236
rs3026101	+	-	-	17	5280440	*RABEP1*	-0.079	0.042	0.057	-0.065	0.031	0.039	-0.001	0.001	0.304
rs4925114	+	-	-	17	17711270	*RAI1*	-0.057	0.065	0.384	0.010	0.049	0.844	-0.002	0.002	0.333
rs4986044	+	-	-	17	21261560	*MAP2K3, KCNJ12*	-0.030	0.043	0.492	0.002	0.033	0.963	-0.002	0.001	0.221
rs17826219	+	-	-	17	29161845	*ATAD5*	-0.006	0.053	0.906	-0.020	0.040	0.611	0.000	0.002	0.974
rs12150665	+	-	-	17	34914787	*GGNBP2*	-0.070	0.042	0.092	-0.069	0.032	0.030	-0.001	0.001	0.513
rs11651052	+	-	-	17	36102381	*HNF1B*	-0.060	0.044	0.171	0.032	0.033	0.326	-0.002	0.001	0.058
rs11658063	+	-	-	17	36103872	*HNF1B*	-0.060	0.044	0.171	0.031	0.033	0.345	-0.002	0.001	0.059
rs6504108	+	-	-	17	46292923	*SKAP1*	0.060	0.051	0.233	0.044	0.038	0.251	0.002	0.001	0.251
rs11079830	-	+	-	17	46682630	*HOXB-AS3*	-0.030	0.042	0.470	-0.010	0.032	0.759	-0.001	0.001	0.682
rs12602912	+	-	-	17	65870073	*BPTF*	-0.049	0.044	0.262	0.012	0.033	0.709	-0.002	0.001	0.087
rs4790981	+	-	-	17	65921834	*BPTF*	-0.069	0.044	0.122	0.008	0.034	0.805	-0.003	0.001	0.034
rs12940622	+	-	-	17	78615571	*RPTOR*	-0.074	0.043	0.082	-0.003	0.032	0.919	-0.002	0.001	0.113
rs1788820	+	-	-	18	21101944	*C18orf8*	0.154	0.074	0.038	0.021	0.056	0.714	0.006	0.002	0.005
rs9304204	+	-	-	18	36896003	*MIR924HG*	-0.014	0.046	0.765	-0.015	0.035	0.663	0.001	0.001	0.692
rs1518170	+	-	-	18	40708905	*RIT2, SYT4*	0.039	0.044	0.377	0.037	0.033	0.265	0.001	0.001	0.485
rs1518142	+	-	-	18	40721490	*RIT2, SYT4*	0.042	0.044	0.337	0.032	0.033	0.333	0.001	0.001	0.385
rs4129322	+	-	-	18	50605642	*DCC*	-0.042	0.043	0.335	-0.003	0.033	0.926	-0.002	0.001	0.233
rs7243785	+	-	-	18	52475162	*DYNAP, RAB27B*	-0.040	0.045	0.376	-0.059	0.034	0.083	0.000	0.001	0.783
rs8092503	-	+	-	18	52479487	*DYNAP, RAB27B*	-0.040	0.045	0.374	-0.059	0.034	0.081	0.000	0.001	0.785
rs6567160	+	+	-	18	57829135	*PMAIP1, MC4R*	0.186	0.051	0.000	0.129	0.038	0.001	0.004	0.002	0.019
rs571312	-	-	+	18	57839769	*PMAIP1, MC4R*	0.183	0.051	0.000	0.126	0.038	0.001	0.003	0.002	0.021
rs12454712	+	-	-	18	60845884	*BCL2*	0.001	0.041	0.980	-0.049	0.031	0.114	0.000	0.001	0.764
rs17710386	+	-	-	18	63461201	*CDH7*	0.010	0.043	0.816	0.031	0.032	0.335	-0.001	0.001	0.640
rs183353539	-	-	+	19	6992244	*ADGRE4P, FLJ25758*	0.082	0.059	0.161	0.079	0.044	0.073	0.001	0.002	0.415
rs17724992	+	-	-	19	18454825	*PGPEP1*	0.022	0.041	0.592	-0.006	0.031	0.848	0.001	0.001	0.546
rs8105895	-	-	+	19	22215457	*ZNF208, ZNF257*	0.038	0.071	0.591	0.000	0.054	0.996	0.002	0.002	0.429
rs4805566	+	-	-	19	30918316	*ZNF536*	0.079	0.049	0.105	-0.010	0.037	0.793	0.002	0.001	0.126
rs29941	+	-	-	19	34309532	*KCTD15, LSM14A*	0.045	0.050	0.369	0.069	0.038	0.071	0.001	0.001	0.587
rs11671664	+	-	-	19	46172278	*GIPR*	0.121	0.041	0.003	0.055	0.031	0.076	0.002	0.001	0.076
rs35560038	+	-	-	19	46175046	*GIPR*	0.142	0.042	0.001	0.060	0.032	0.059	0.003	0.001	0.013
rs1884897	+	-	-	20	6612832	*CASC20, LINC01713*	0.028	0.071	0.696	-0.105	0.054	0.051	0.003	0.002	0.234
rs677673	-	-	+	20	10481772	*SLX4IP*	0.013	0.044	0.768	-0.029	0.033	0.379	0.000	0.001	0.857
rs16978956	+	-	-	20	18288165	*ZNF133*	0.039	0.052	0.453	0.065	0.039	0.099	0.000	0.002	0.962
rs7274963	+	-	-	20	18290979	*ZNF133*	0.036	0.052	0.492	0.060	0.039	0.128	0.000	0.002	0.961
rs11698834	-	-	+	20	23832595	*CST2, CST5*	0.024	0.043	0.576	0.007	0.032	0.836	0.000	0.001	0.840
rs6091540	+	-	-	20	51087862	*ZFP64, LINC01524*	-0.036	0.042	0.400	0.034	0.032	0.294	-0.002	0.001	0.097
rs2247627	+	-	-	20	54145086	*LINC01441, CBLN4*	0.027	0.041	0.502	0.017	0.031	0.576	0.000	0.001	0.719
rs2243930	+	-	-	20	54147462	*LINC01441, CBLN4*	0.042	0.041	0.311	0.016	0.031	0.618	0.001	0.001	0.447
rs6089584	+	-	-	20	60564086	*TAF4*	-0.028	0.043	0.514	0.002	0.032	0.962	0.000	0.001	0.836
rs2836768	+	-	-	21	40311493	*LOC400867, LINC01700*	0.110	0.048	0.023	0.074	0.037	0.044	0.002	0.001	0.268
rs9983113	+	-	-	21	40315316	*LOC400867, LINC01700*	0.101	0.048	0.034	0.068	0.036	0.057	0.001	0.001	0.310
rs427943	+	-	-	21	46570896	*ADARB1*	0.013	0.041	0.760	-0.043	0.031	0.163	0.001	0.001	0.384
rs4820408	+	-	-	22	40604945	*TNRC6B*	-0.030	0.041	0.459	-0.060	0.031	0.053	0.000	0.001	0.941
rs139913	+	-	-	22	40713861	*TNRC6B*	-0.023	0.041	0.568	-0.053	0.031	0.086	0.000	0.001	0.848

BMI, body mass index; bp, base pair; Chr., chromosome; SE, standard error; SNP, single nucleotide polymorphism.

We extracted candidate SNPs which marked "+" [a]from the BioBank Japan project report, by searching [b] "childhood body mass index" and [c] "longitudinal BMI measurement" in the GWAS catalog.

[d]Positions are based on Human Genome version 19 (hg19), build 37. [e]Alternative alleles were treated as effect alleles.

[f]Significant *P* -values were defined as $P \le 1.77E\text{-}04$.

[g]Adjusted models were adjusted for age (continuous), sex (male or female), site (12 areas), and birth year (continuous).

eTable 2. List of SNPs excluded from the initial candidate variants for analysis

SNP	Current BMI[a]	BMI at 20[b]	BMI change[c]	Chr.[d]	Position (bp)[d]	Candidate gene(s)
(i) No available in imputed genotyping data						
rs17264034			+	5	9557602	*AC026787.1,SNHG18*
rs6885147			+	5	64448670	*ADAMTS6*
rs4869139	+			5	95858668	*LOC101929710*
rs4308481	+			5	122652106	*PRDM6,CEP120*
rs3849724	+			5	173290977	*LINC01485,CPEB4*
rs183975233	+			6	32437160	*HLA-DRA,HLA-DRB5*
rs115472351	+			6	32591448	*HLA-DRB1,HLA-DQA1*
rs10120246			+	9	9799050	*PTPRD*
rs11607976	+			11	69279111	*MYEOV,LINC01488*
rs180950758	+			17	29036425	*SUZ12P1*
rs1379871	+			X	31854782	*DMD*
rs6529684	+			X	53542107	*HSD17B10,HUWE1*
rs3121672	+			X	117916370	*IL13RA1*
rs1190736	+			X	136113464	*GPR101*
rs5945324	+			X	152894551	*FAM58A,DUSP9*
(ii) MAF <0.05						
rs2590942		+		1	72885281	*RNU6-1246P,RPL31P12*
rs7550711		+		1	110082886	*AL355310.3,GPR61*
rs17024393		+		1	110154688	*GNAT2,GNAI3*
rs199950			+	1	181590858	*CACNA1E*
rs34585985			+	1	210489411	*AL035414.1,HHAT*
rs10208649	+			2	54161363	*PSME4*
rs13387838		+		2	207281447	*C017081.1,HNRNPA1P51*
rs7599312	+			2	213413231	*ERBB4,MIR4776-2*
rs41526344			+	3	2985142	*CNTN4*
rs1899951	+			3	12394840	*PPARG*
rs9847124			+	3	152590762	*HMGN2P13,P2RY1*
rs1516725	+			3	185824004	*ETV5*
rs6447650			+	4	40381210	*RNU7-74P,RBM47*
rs13107325		+		4	103188709	*SLC39A8*
rs28580297			+	4	119427957	*CEP170P1*
rs25832		+		5	66175682	*MAST4*
rs10041997			+	5	120198311	*AC114284.1,AC008565.1*
rs17107133			+	5	147059015	*JAKMIP2*
rs7715256		+		5	153537893	*MFAP3*
rs4569924		+		5	153540025	*MFAP3*
rs1347155			+	5	171969348	*SH3PXD2B,LINC01944*
rs148546399	+			6	64705610	*EYS*
rs17601580			+	6	132061420	*ENPP3*
rs1167827	+			7	75163169	*HIP1*
rs7869969		+		9	96217447	*FAM120A*
rs7903146	+			10	114758349	*TCF7L2*
rs4751240			+	10	129136409	*DOCK1*
rs17309930		+		11	27748493	*AC103796.1*
rs12865097			+	13	45477410	*LINC00330,NUFIP1*
rs10151686		+		14	30466466	*AL133372.2,PRKD1*
rs927251			+	14	58311135	*SLC35F4*
rs12897644			+	14	95838401	*SYNE3,LINC02292*
rs12446632		+		16	19935389	*GPR139,GPRC5B*
rs55769859			+	22	46353046	*WNT7B*

BMI, body mass index; bp, base pair; Chr., chromosome; MAF: minor allele frequency; SE, standard error; SNP, single nucleotide polymorphism.

We extracted candidate SNPs which marked "+" [a]from the BioBank Japan project report, by searching [b] "childhood body mass index" and [c] "longitudinal BMI measurement" in the GWAS catalog.

[d]Positions are based on Human Genome version 19 (hg19), build 37.

eTable 3. Sex-stratified analysis of the 11 significant SNPs

SNP	Chr.[a]	Position (bp)[a]	REF/ALT	Candidate gene(s)	Current BMI							BMI at 20							BMI change						
					Male			Female			*P* -heterogeneity by sex[g]	Male			Female			*P* -heterogeneity by sex[g]	Male			Female			*P* -heterogeneity by sex[g]
					Adjusted[d]			Adjusted[d]				Adjusted[e]			Adjusted[e]				Adjusted[f]			Adjusted[f]			
					β[b]	SE	*P* -value[c]	β[b]	SE	*P* -value[c]		β[b]	SE	*P* -value[c]	β[b]	SE	*P* -value[c]		β[b]	SE	*P* -value[c]	β[b]	SE	*P* -value[c]	
rs939584	2	621558	C/T	*LOC105373352, TMEM18*	-0.206	0.097	3.43E-02	-0.228	0.092	1.30E-02	0.918	-0.297	0.077	1.16E-04	-0.144	0.067	3.10E-02	0.127	0.000	0.003	9.66E-01	-0.005	0.003	7.12E-02	0.441
rs13021737	2	632348	A/G	*LOC105373352, TMEM18*	-0.186	0.097	5.51E-02	-0.232	0.091	1.11E-02	0.914	-0.269	0.077	4.88E-04	-0.154	0.067	2.07E-02	0.260	0.000	0.003	9.64E-01	-0.005	0.003	7.14E-02	0.387
rs4854349	2	647861	T/C	*LOC105373352, TMEM18*	-0.170	0.097	7.83E-02	-0.204	0.091	2.46E-02	0.984	-0.257	0.077	8.20E-04	-0.131	0.066	4.80E-02	0.215	0.000	0.003	9.33E-01	-0.004	0.003	1.07E-01	0.442
rs4409766	10	104616663	T/C	*BORCS7-ASMT*	0.176	0.066	7.39E-03	0.201	0.060	7.94E-04	0.734	0.061	0.052	2.41E-01	0.022	0.044	6.17E-01	0.501	0.004	0.002	1.88E-02	0.006	0.002	8.88E-04	0.543
rs11030100	11	27677586	G/T	*BDNF-AS*	-0.192	0.061	1.68E-03	-0.206	0.056	2.14E-04	0.382	-0.100	0.049	3.86E-02	-0.063	0.041	1.19E-01	0.588	-0.004	0.002	2.89E-02	-0.005	0.002	1.38E-03	0.201
rs6265	11	27679916	C/T	*BDNF*	-0.189	0.061	1.97E-03	-0.218	0.056	8.86E-05	0.252	-0.093	0.048	5.38E-02	-0.051	0.041	2.12E-01	0.555	-0.004	0.002	2.39E-02	-0.006	0.002	3.31E-04	0.112
rs11030104	11	27684517	A/G	*BDNF-AS*	-0.189	0.061	2.00E-03	-0.219	0.056	8.34E-05	0.236	-0.089	0.049	6.59E-02	-0.047	0.041	2.45E-01	0.580	-0.004	0.002	2.13E-02	-0.006	0.002	2.27E-04	0.099
rs1927790	13	96922191	T/C	*HS6ST3*	0.102	0.061	9.26E-02	0.026	0.055	6.39E-01	0.302	0.131	0.048	6.67E-03	0.112	0.040	5.30E-03	0.657	0.001	0.002	6.19E-01	-0.001	0.002	6.02E-01	0.361
rs1421085	16	53800954	T/C	*FTO*	0.264	0.075	4.39E-04	0.390	0.069	1.91E-08	0.070	0.159	0.060	7.87E-03	0.184	0.051	2.73E-04	0.805	0.005	0.002	1.62E-02	0.008	0.002	1.24E-04	0.135
rs11642015	16	53802494	C/T	*FTO*	0.262	0.075	5.04E-04	0.399	0.069	8.21E-09	0.053	0.159	0.060	7.99E-03	0.184	0.051	2.79E-04	0.810	0.005	0.002	1.80E-02	0.009	0.002	4.85E-05	0.095
rs1558902	16	53803574	T/A	*FTO*	0.265	0.075	4.35E-04	0.394	0.069	1.34E-08	0.064	0.159	0.060	7.77E-03	0.184	0.051	2.74E-04	0.809	0.005	0.002	1.66E-02	0.008	0.002	9.26E-05	0.121

ALT, alternative allele; BMI, body mass index; bp, base pair; Chr., chromosome; REF, reference allele; SE, standard error; SNP, single nucelotide polymorphism.

[a]Positions are based on Human Genome version 19 (hg19), build 37. [b]Alternative alleles were treated as effect alleles.

[c]Significant *P* -values were defined as *P* ≤1.77E-04.

[d]Adjusted model in current BMI were adjusted for age (continuous), age-squared (continuous), birth year (continuous), and the first five principal components (continuous).

[e]Adjusted model in BMI at 20 were adjusted for birth year (continuous), and the first five principal components (continuous).

[f]Adjusted model in BMI change were adjusted for age (continuous), age-squared (continuous), birth year (continuous), the first five principal components (continuous), and BMI at 20 (continuous).

[g]Significant heterogeneity *P* -values were defined as *P* ≤0.05

eTable 4. Analysis of 11 significant SNPs excluding DM population in current BMI

SNP	Chr.[a]	Position (bp)[a]	REF/ALT	Candidate gene(s)	Current BMI: Whole population, Adjusted model[d] β[b]	SE	*P*-value[c]	Current BMI: Excluding DM population, Adjusted model[d] β[b]	SE	*P*-value[c]
rs939584	2	621558	C/T	*LOC105373352, TMEM18*	-0.220	0.067	1.07E-03	-0.201	0.068	3.07E-03
rs13021737	2	632348	A/G	*LOC105373352, TMEM18*	-0.214	0.067	1.48E-03	-0.194	0.068	4.25E-03
rs4854349	2	647861	T/C	*LOC105373352, TMEM18*	-0.191	0.067	4.26E-03	-0.170	0.068	1.20E-02
rs4409766	10	104616663	T/C	*BORCS7-ASMT*	0.181	0.045	**4.96E-05**	0.168	0.045	1.91E-04
rs11030100	11	27677586	G/T	*BDNF-AS*	-0.199	0.042	**1.68E-06**	-0.191	0.042	**5.03E-06**
rs6265	11	27679916	C/T	*BDNF*	-0.203	0.041	**1.00E-06**	-0.194	0.042	**3.33E-06**
rs11030104	11	27684517	A/G	*BDNF-AS*	-0.203	0.042	**1.01E-06**	-0.193	0.042	**3.93E-06**
rs1927790	13	96922191	T/C	*HS6ST3*	0.057	0.041	1.64E-01	0.078	0.042	5.95E-02
rs1421085	16	53800954	T/C	*FTO*	0.335	0.051	**1.24E-10**	0.318	0.052	**1.12E-09**
rs11642015	16	53802494	C/T	*FTO*	0.334	0.051	**7.36E-11**	0.322	0.052	**6.89E-10**
rs1558902	16	53803574	T/A	*FTO*	0.331	0.051	**8.65E-11**	0.320	0.052	**8.17E-10**

ALT, alternative allele; BMI, body mass index; bp, base pair; Chr., chromosome; DM, diabetes mellitus; REF, reference allele; SE, standard error; SNP, single nucleotide polymorphism.

[a]Positions are based on Human Genome version 19 (hg19), build 37. [b]Alternative alleles were treated as effect alleles. [c]Significant *P*-values ($P \leq 1.77E\text{-}04$) are shown in bold.

[d]Adjusted model in current BMI were adjusted for age (continuous), age-squared (continuous), sex (male or female), birth year (continuous), and the first five principal components (continu

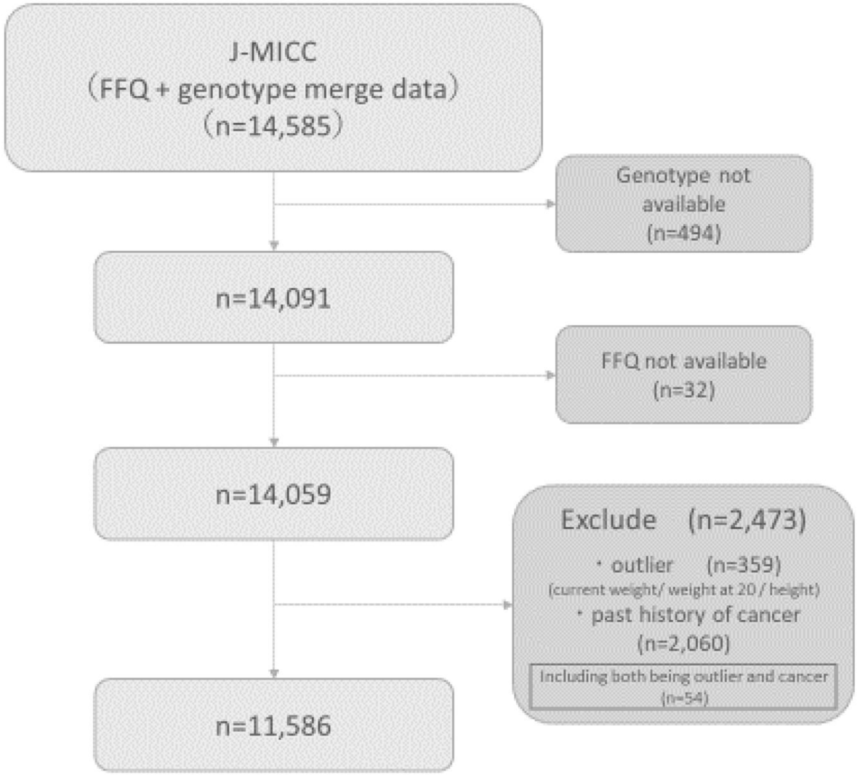

eFigure 1. Consort diagram of the eligibility of subjects after merging questionnaire and genotype data. FFQ, food frequency questionnaire.

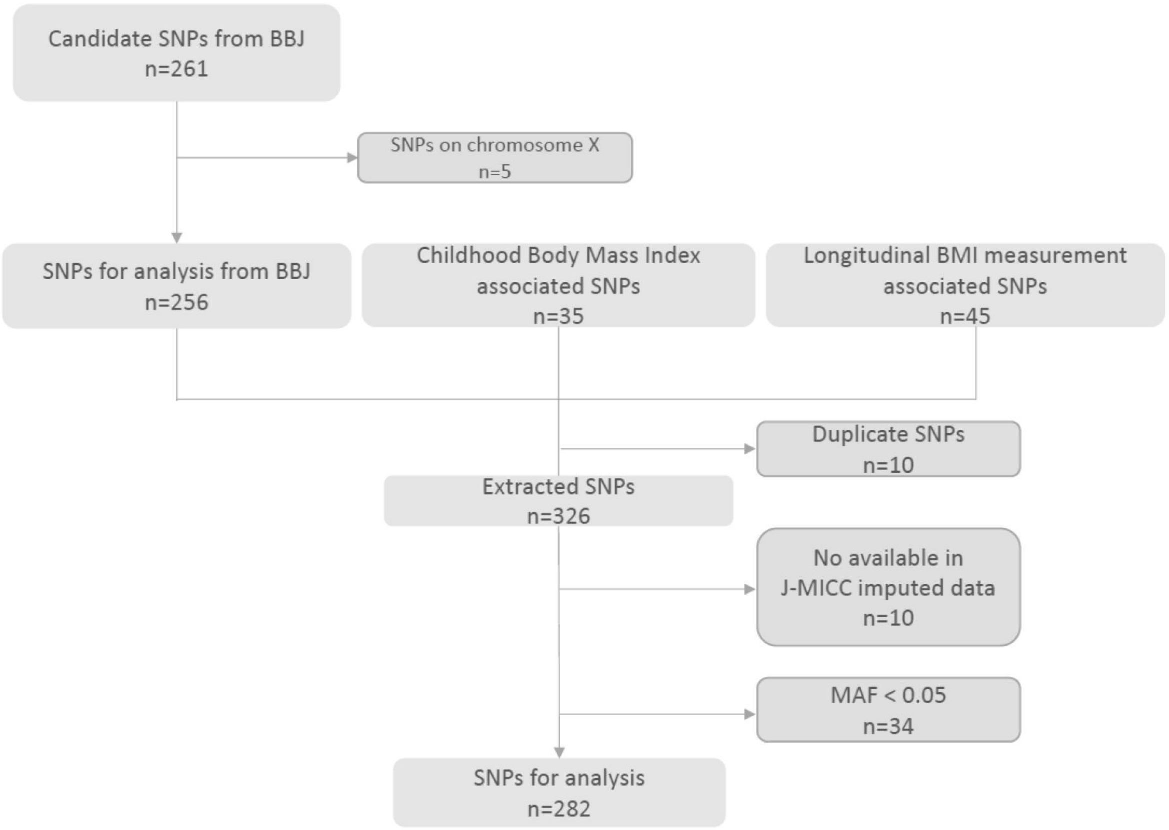

eFigure 2. Consort diagram of the selection of candidate loci. BBJ, BioBank Japan study[15]; BMI, body mass index; MAF, minor allele frequency; SNPs, single nucleotide polymorphisms.
